# Comparison of the Effects between Tannins Extracted from Different Natural Plants on Growth Performance, Antioxidant Capacity, Immunity, and Intestinal Flora of Broiler Chickens

**DOI:** 10.3390/antiox12020441

**Published:** 2023-02-10

**Authors:** Song Liu, Kaijun Wang, Senzhu Lin, Zhidan Zhang, Ming Cheng, Shanshan Hu, Hongjie Hu, Jun Xiang, Fengming Chen, Gonghe Li, Hongbin Si

**Affiliations:** 1 State Key Laboratory for Conservation and Utilization of Subtropical Agro-Bioresources, College of Animal Science and Technology, Guangxi University, Nanning 530004, China; 2 Animal Nutritional Genome and Germplasm Innovation Research Center, College of Animal Science and Technology, Hunan Agricultural University, Changsha 410125, China; 3 Academician Workstation, Changsha Medical University, Changsha 410028, China

**Keywords:** tannin, broilers, antioxidant, gut microbiota, immunity, anti-inflammatory

## Abstract

**Simple Summary:**

Tannins are widely found in natural plants and have a wide range of biological activities. Here, we compared the effects of different sources of tannins on broilers and found that tannins from different sources have their own unique properties, so each tannin can have a significant impact on the growth of broilers. *Acacia mearnsii* tannin extract has a positive effect on the nutrient absorption of broilers, which can increase the abundance of *Lactobacillus* in the intestine and enhance nutrient absorption; *Castanea sativa* tannin has excellent antioxidant and anti-inflammatory properties and can be used as immune enhancement additives, but it has no effect on the digestion and absorption capacity of broilers; both *Schinopsis lorenzii* tannin extract and *Caesalpinia spinosa* tannin extract have significant hepatoprotective effects, so these two tannins can be used for liver protection in broilers.

**Abstract:**

In this study, four plant tannins, including AT (*Acacia mearnsii* tannin, 68%), CT (*Castanea sativa* tannin, 60%), QT (*Schinopsis lorenzii* tannin, 73%) and TT (*Caesalpinia spinosa* tannin, 50%) were added to broiler diets for 42 days to evaluate and compare their effects on growth performance, antioxidant capacity, immune performance and gut microbiota in broilers. The results showed that the supplementation of five tannins could increase the production of T-AOC, GSH-Px, SOD and CAT and reduce the production of MDA in the serum of broilers (*p* < 0.01), but the antioxidant effect of the AT group was lower than that of the other three groups (*p* < 0.01). All four tannins decreased the level of the pro-inflammatory factor IL-1β and increased the level of the anti-inflammatory factor IL-10 (*p* < 0.01). CT, QT and TT decreased the levels of pro-inflammatory factors IL-6 and TNF-α (*p* < 0.01), while AT and CT increased the level of IL-2 in serum (*p* < 0.01). Supplementation with four tannins also increased the levels of IgG, IgM, IgA and sIgA in serum (*p* < 0.01) and the levels of ZO-1, claudin-1 and occludin in the jejunum (*p* < 0.01). The detection results of ALT and AST showed that CT, QT and TT decreased the concentrations of ALT and AST in serum (*p* < 0.01). The results of the gut microbiota showed that the abundance of *Clostridia* and *Subdoligranulum* increased, and the abundance of *Oscillospiraceae* decreased, compared to the control group after adding the four tannins to the diets (*p* > 0.05). In addition, CT, QT and TT decreased the abundance of *Lactobacillus* and increased the abundance of *Bacteroides* compared to the control group, while AT showed the opposite result (*p* > 0.05). Overall, our study shows that tannins derived from different plants have their own unique effects on broilers. AT and CT can promote broilers’ growth better than other tannins, CT has the best ability to improve immune and antioxidant properties, and QT and TT have the best effect on broilers’ liver protection.

## 1. Introduction

For decades, antibiotics have been widely used as feed additives to treat and improve animal health and enhance their biological functions, growth performance and utilization of feed nutrients [1]. However, what followed was the occurrence of various types of bacterial resistance [2], and the abuse of antibiotics has led to the emergence of multi-drug resistant bacteria, including vancomycin-resistant *Enterococcus* (VRE), Methicillin-resistant *Staphylococcus aureus* (MRSA) and carbapenem-resistant *Enterobacteriaceae* (CRE), which seriously threaten animal and human health [3].

Fortunately, several studies in recent years identified additives with the same beneficial effects as antibiotics, such as prebiotics, and botanical additives [4]. Botanical additives include essential oils, plant extracts, plant secondary metabolites, etc. They have attracted a lot of attention from researchers for their positive impact on the improvement of livestock productivity and widespread existence in nature, making them a natural alternative to antibiotics [5,6]. Phenolic compounds isolated from plants have strong antioxidant properties as well as additional advantageous properties such as antibacterial, anti-inflammatory, and enhancement of intestinal immunity, so they are considered to be effective feed additives and have become the focus of animal scientists [7].

Tannins (TA) are natural phenolic compounds widely present in nature [8], which can be extracted from the fruits, bark, leaves, flowers and other parts of most plants [9]. The structures of tannins are diverse and complex. They are divided into *hydrolyzable tannins* (HT), *condensed tannins* (CT) and *phlorotannins* (PT) according to their structures [10]. HT are an ester composed of phenolic acid or its derivatives combined with glucose or polyol, which can be hydrolyzed into polyol and phenolic acid by acids and bases [11]. The molecular weights are relatively low, ranging from 500 to 3000 Da generally. CT, commonly known as proanthocyanidins, are a polymer formed by the polymerization of flavan-3-ol and flavan-3,4-diol, which are condensed through oxidative dehydration caused by acids, bases and bacteria. Compared to HT, CT have higher molecular weights, ranging from 1000 to 20,000 Da [12]. Finally, PT are a polyphenol extracted from brown seaweed, synthesized from phloroglucinol via the acetate–malonate pathway, with molecular weights ranging from 126 Da to 650 kDa [13].

Many studies have evaluated the biological functions of the purified tannins or tannins extracted from natural plants in in vivo and in vitro animal models [14,15,16,17,18]. The addition of low-dose tannins to silage can improve the use efficiency of nitrogen and methane emission reduction in ruminants without adversely affecting rumen fermentation and gut microbiota [15]. In addition, this is beneficial for piglets with post-weaning diarrhea (PWD), as it improves their growth performance and regulates the intestinal flora to maintain intestinal health, when tannins extracted from chestnuts are added [16]. Similarly, adding gallic tannins to poultry diets has an anticoccidial effect and a regulating effect on the intestinal flora of chickens [17], while adding chestnut tannins can improve the antioxidant properties and cholesterol metabolism of broilers and can promote growth, without affecting the normal meat quality of broilers [18].

It is obvious that the effects of tannins extracted from various plants on livestock have differences from the above studies. Hence, comparing the features of tannins extracted from different natural plants on livestock allows us to understand the biological functions of them, which helps us to conduct targeted research on the functions of different tannins in the future. With the further development of sequencing technology, we can explore the impact of animal diet changes on the gut microbiome [19,20,21]. In addition, comparative studies on various tannins have, so far, only been reported on lambs, while there are no related studies on poultry [22]. Therefore, this study compared the effects of tannins extracted from four different natural plants on the growth performance, biological functions, intestinal flora and metabolism of broiler chickens.

## 2. Materials and Methods

### 2.1. Animals and Treatment

The animal experiments were carried out in accordance with the Animal Care and Use Committee of Guangxi University, Nanning, China (Gxu-2022-262). A total of 100 one-day-old AA broiler chicks were purchased from a local commercial producer. The broilers in the experiment were all male broilers. Adopting the method of flat rearing, the size of pen was 160 cm × 60 cm × 100 cm. The broiler house was well-ventilated and well-lighted, and the relative humidity was maintained at 55% to 65%. The broilers were exposed to 24 h of light at the age of 1 to 14 days, which was then reduced to 20 h of light per day. The temperature was kept at 32 to 34 °C for the first week, then it was gradually reduced by 1 °C every 3 days until the final temperature was 24 to 26 °C. During the whole experiment, the chickens had free access to water and food. Broilers were vaccinated on the first day. Before starting the experiment, broilers were submitted to a 7-day adaptation period, and they were fed a basal diet during this period. Then, they were weighed and randomly divided into six treatments on the seventh day. Each treatment contained five replicates, and there were four broilers per replicate. The average weight was 0.16 kg ± 0.01 per treatment. The control group was fed the basal diet, and the other five groups were fed the basal diet with added tannins extracted from different natural plants in the basal diet, from the eighth day to the end of the experiment. The added tannins were *Acacia mearnsii* tannin extract (68%, AT), *Castanea sativa* tannin (60%, CT), *Schinopsis lorenzii* tannin extract (73%, QT) and *Caesalpinia spinosa* tannin extract (50%, TT), provided by Silvateam.s.p.a, SanMicheleMondovì, Italy. All tannin extracts were added to basal diets at a concentration of 600 mg/kg. Among them, AT and QT are condensed tannins, while CT and TT are hydrolyzed tannins. The information of the diet is shown in Table 1.

### 2.2. Growth Performance

Broilers were weighed on the seventh day to obtain the average initial body weight, and then body weights were recorded on days 7, 14, 21, 28, 35, 42 and 49 to calculate average daily gain (ADG). Feed consumption was recorded weekly to calculate average daily feed intake (ADFI). Feed conversion rate (FCR) was recorded as ADFI:ADG. In addition, the diarrhea rates of each replicate were recorded daily.

### 2.3. Sample Collection

At the end of feeding period (49 days), 2 broilers were randomly selected from each replicate, which means 10 broilers per group. Broiler blood was collected by wing vein blood collection. Then, serum was centrifuged at 3000 rpm at 4 °C and stored at −20 °C for biochemical analysis [23]. Next, broilers were anesthetized and sacrificed. The jejunum was harvested and fixed in 10% formalin for histomorphological examination [24]. Liver tissue was collected for biochemical analysis following the method of Ma et al. [25]. The jejunal mucosa was scraped with a glass microscope slide to measure intestinal tight junction protein. The cecum was taken out, the length of it was measured, and then cecal contents were collected for gut microbiota analysis. All samples were stored at −80 °C until assayed.

### 2.4. Measurement of Antioxidant Activity, Immune Factors and Liver Function

ELISA kits (BYabscience Biotechnelogy Co., Ltd., Nanjing, China) were used to measure the levels of antioxidative activity-related factors (T-AOC, MDA, SOD, GSH-Px and CAT), inflammation-related factors (IL-1β, IL-2, IL-6, IL-10, IL-21 and TNF-α) and immunoglobulin (IgG, IgM, IgA and sIgA) in serum. In addition, we also measured the concentration of ALT and AST in serum by the same method.

### 2.5. Measurement of Intestinal Tight Junction Protein

In accordance with the instructions of the ELISA kit (BYabscience Biotechnelogy Co., Ltd., Nanjing, China), the jejunal mucosa was homogenized and then sonicated. Finally, the homogenate was centrifuged at 5000 rpm for 5–10 min, and the supernatant was taken for measuring the levels of zonula occluden-1 (ZO-1), claudin-1 and occludin.

### 2.6. Histological Examination

The jejunum samples were fixed in 10% neutral formalin for 72 h, then processed for dehydration and clearing and embedded in paraffin. Sections (5 μm) were stained with hematoxylin and eosin (H&E) to observe histological changes. Images were acquired with an Eclipse Ci-L photomicroscope (Nikon, Japan) (40× magnification). Intestinal villus height (VH) was measured (µm) from the tip to the base of the villus, and the crypt depth (CD) was calculated from the villus crypt junction to the distal limit of the crypt [26]. The jejunum tissue of 4 broilers in each group was selected as slices, 3 fields of view were selected from each jejunal tissue section, and 10 villus heights and 10 crypt depths were measured in each field with Image-Pro Plus 6.0. Finally, the average value and the ratio of villi height to crypt depth were calculated to assess the digestive and absorptive capacity of the intestine.

### 2.7. Gut Microbiome Analysis

DNA from cecal contents was extracted with the E.Z.N.A.^®^ soil DNA Kit (Omega Bio-Tek, Norcross, GA, USA) and detected by 1% agarose gel electrophoresis [27]. The V3-V4 hypervariable regions of the bacterial 16S-rDNA gene were amplified by universal primers 341F (5′-CCTAYGGGRBGCASCAG-3′) and 806R (5′-GGACTACNNGGGTATCTAAT-3′). PCR was performed in a 20 μL mixture, and all products were purified by AxyPrep DNA Gel Recovery kit (Axygen, Union City, NJ, USA) and quantified by QuantiFluor™ -ST (Promega, Madison, WI, USA). PE library was built up using TruSeCT60M DNA Sample Prep Kit and sequenced by an Illumina Miseq PE300 platform (Illumina, San Diego, CA, USA) according to the standard scheme [28]. High quality sequence clustering was performed using UPARSE software to obtain Operational Taxonomic Units (OTUs) based on 97% similarity. Alpha diversity and beta diversity were analyzed according to the abundances of OTUs using the R package. The relative abundance of the dominant bacteria at phylum and genus levels were analyzed. Genus level classifications of bacteria were compared with the Wilcoxon rank-sum test. The one-way ANOVA test was used for multiple comparisons, and the Wilcoxon rank-sum test was used for two-group comparisons.

### 2.8. Statistical Analysis

All of the experimental data in the tables and figures are presented as mean ± SD, and the differences between groups were assessed by one-way ANOVA and Tukey’s multiple comparisons using SPSS (IBM SPSS 21.0, Chicago, IL, USA). Graphs were made by GraphPad Prism 6.0. The data of 16S rRNA sequencing were analyzed on the Majorbio cloud platform. Correlation coefficients were performed between antioxidant activity-related factors, differential microbes, body weight, immune factors (immunoglobulins and inflammatory cytokines) and levels of gut tight junction proteins. Correlation coefficients were calculated using Spearson correlation analysis by the R package to identify bivariate relationships between variables. Significant differences were identified at *p* < 0.05, and markedly significant differences were identified at *p* < 0.01.

## 3. Results

### 3.1. Growth Performance

The broiler house was disinfected regularly during the experiment, and no adverse events occurred. The effects of the diets that were supplemented with tannins on broilers’ weight are shown in Table 2. Compared with the control group, the diets supplemented with different tannins had no significant effect on the body weight of broilers, and there were no statistical differences between the groups. The comparison between experimental groups showed that the body weight of AT and QT was larger than that of CT in the pre-growth stage (7–28 days), while the body weight of CT was larger than that of AT and QT in the late growth stage (28–49 days), and TT had the smallest body weight throughout the whole growing period compared with the other three groups.

As shown in Table 3, the ADFI, ADG and FCR of each experimental group were not significantly different from CON. However, except for the TT group, the FCR of the other three treatment groups had a downward trend compared with CON. There was no significant difference in ADFI, ADG and FCR between the experimental groups (*p* > 0.05), either. The FCR of CT was lower than that of the other experimental groups in the growth stage.

### 3.2. Diarrhea Rate and Cecum Length

As shown in Table 4, the diarrhea rates of CT, QT and TT showed a downward trend compared with CON, while AT showed an upward trend, but the difference between the experimental groups and CON is not statistically significant. However, the comparison between the treatment groups found that the diarrhea rate of QT is the lowest compared with other three groups, and AT is the highest compared with the other three groups. In addition, by measuring the length of the cecum, we found that the length of the cecum between the CON group and each experimental group was not significantly different after adding tannins to the diet, but the experimental groups all showed an upward trend compared with the CON group. In conclusion, the addition of tannins from different plants to the diet had no significant effect on the diarrhea rate or cecum length of broilers; however, the length of the cecum in broilers showed an upward trend after feeding tannins, and the diarrhea rate in broilers treated with CT, QT and TT showed a downward trend.

### 3.3. Comparative Assessment of Antioxidant Capacity

**Figure 1:**
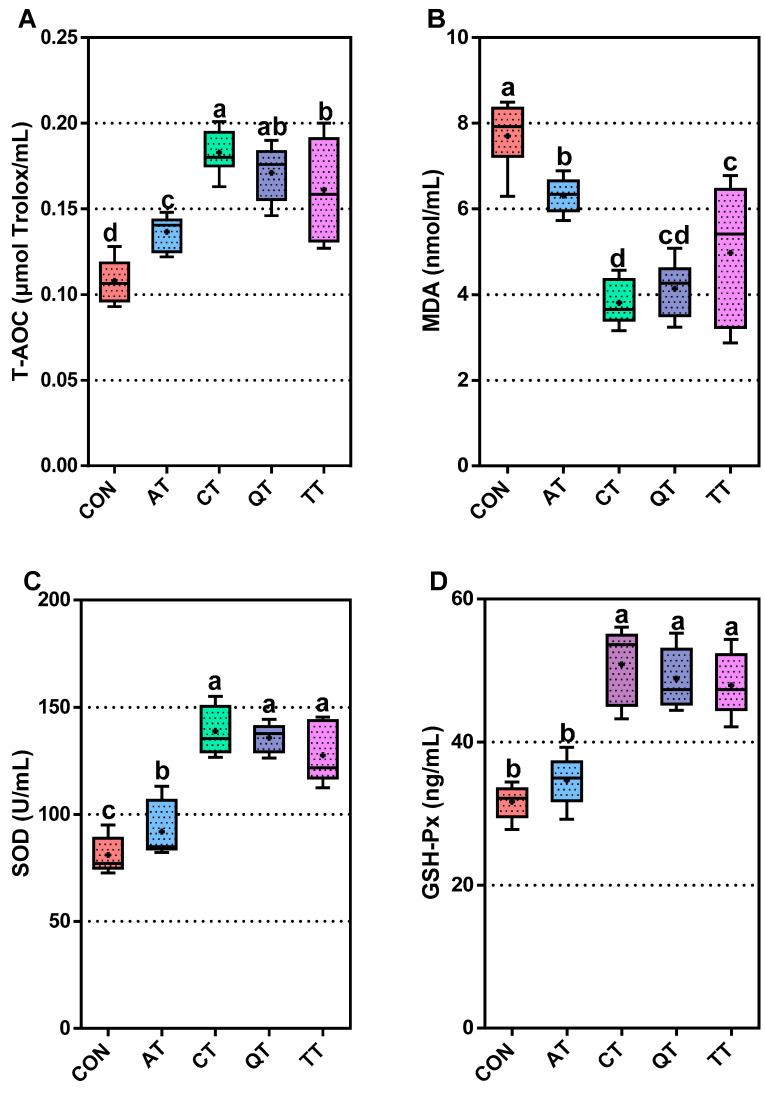

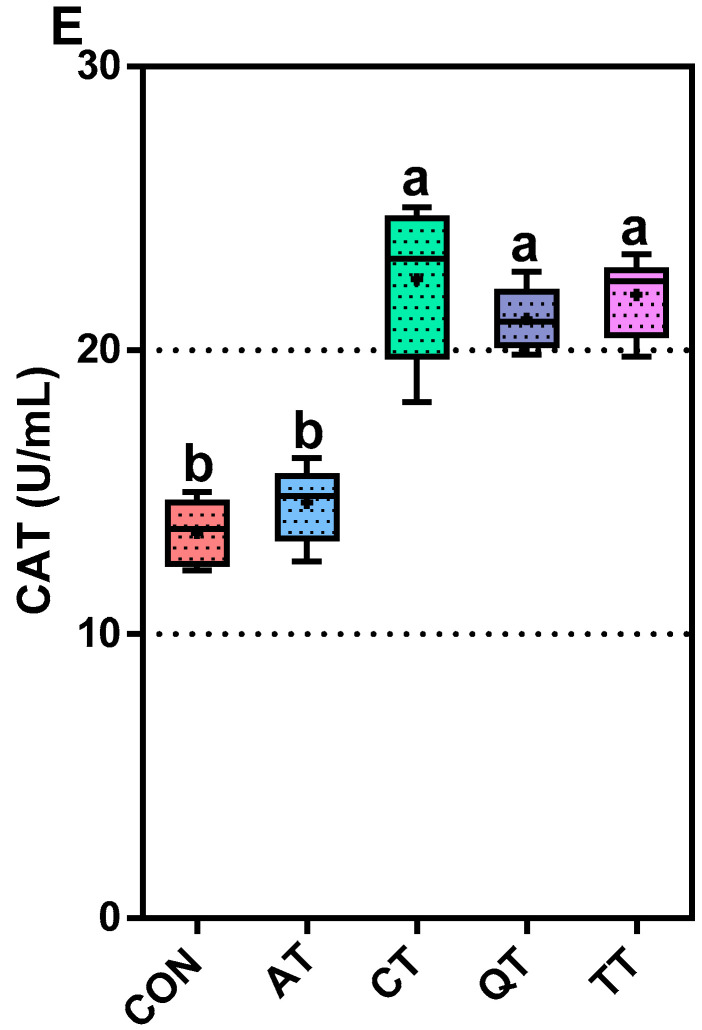
Effects of adding different tannins to the diet on the antioxidant capacity of broilers. (**A**) Total antioxidant capacity, T-AOC; (**B**) malondialdehyde, MDA; (**C**) super oxide dimutese, SOD; (**D**) glutathione peroxidase, GSH-Px; (**E**) catalase, CAT. Different letters (a, b, c and d) indicate significant differences (*p* < 0.05), while the same letter indicates no significant differences (*p* > 0.05). CON, control; AT, *Acacia mearnsii* tannin; CT, *Castanea sativa* tannin; QT, *Schinopsis lorenzii* tannin; TT, *Caesalpinia spinosa* tannin.

To evaluate the effect of the tannins extracted from four natural plants on the antioxidant capacity of broilers, we measured the levels of T-AOC, MDA, SOD, GSH-Px and CAT in serum. The T-AOC in all the treatment groups was significantly higher than CON (*p* < 0.01) (Figure 1A), and their MDA levels were significantly lower than CON (*p* < 0.01) (Figure 1B). The levels of SOD, GSH-Px and CAT in the serum of the CT, QT and TT groups were significantly higher than those of CON (*p* < 0.01) (Figure 1C–E). In addition, AT could significantly increase the level of SOD in serum (*p* < 0.05) compared with CON, but the differences in the GSH-Px and CAT levels between AT and CON was not statistically significant (*p* > 0.05).

The comparison between the experimental groups found that the levels of T-AOC, SOD, GSH-Px and CAT in the serum of AT were markedly significantly lower than in the other three experimental groups (*p* < 0.01), and the level of MDA was markedly significantly higher than that of the other groups (*p* < 0.01). The T-AOC level of CT was higher than TT, and the level of MDA was lower than TT, both of which were statistically significant (*p* < 0.05).

### 3.4. Comparative Assessment of Immunity

**Figure 2:**
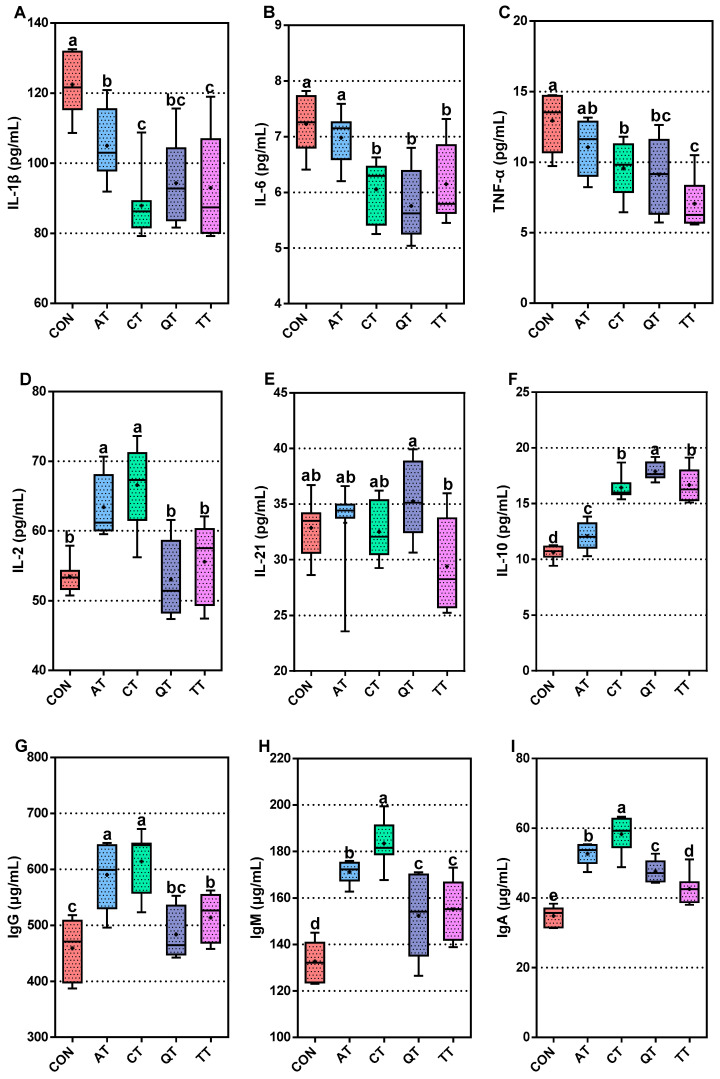

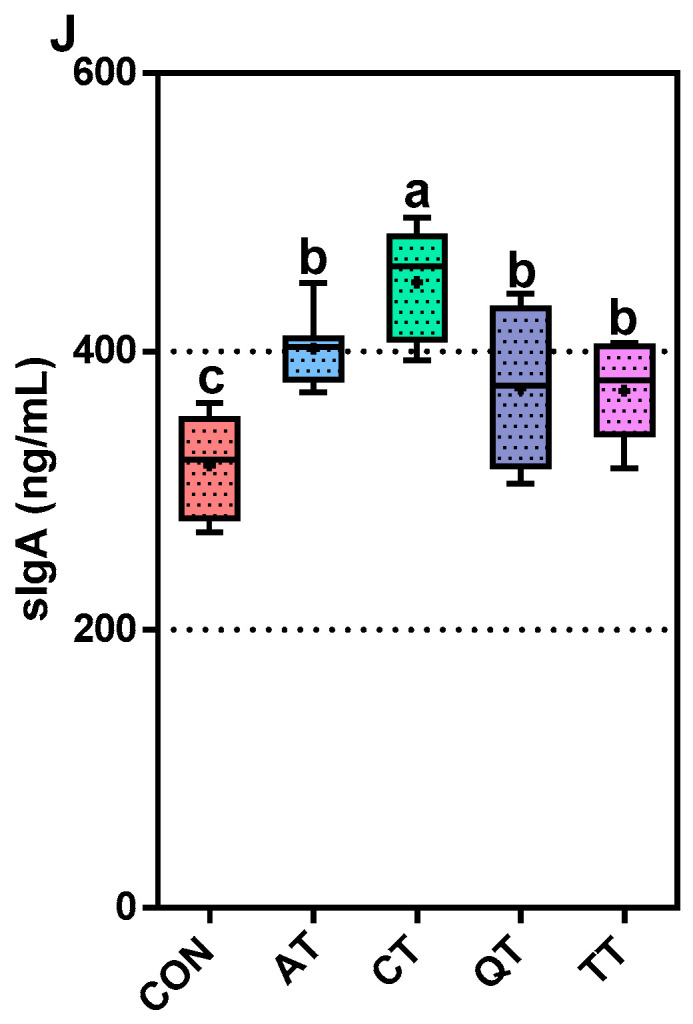
Effects of adding different tannins to the diet on the immunity. (**A**) Interleukin-1β, IL-1β; (**B**) interleukin-6, IL-6; (**C**) tumor necrosis factor-α, TNF-α; (**D**) interleukin-2, IL-2; (**E**) interleukin-21, IL-21; (**F**) interleukin-10, IL-10; (**G**) immunoglobulin G, IgG; (**H**) immunoglobulin M, IgM; (**I**) immunoglobulin A, IgA; (**J**) secretory immunoglobulin A, sIgA. Different letters (a, b, c, d and e) indicate significant differences (*p* < 0.05), while the same letter indicates no significant differences (*p* > 0.05). CON, control; AT, *Acacia mearnsii* tannin; CT, *Castanea sativa* tannin; QT, *Schinopsis lorenzii* tannin; TT, *Caesalpinia spinosa* tannin.

In order to compare the effects of the diets supplemented with different tannins on the immune performance of broilers, we measured the levels of some cytokines, including IL-1β, IL-2, IL-6, IL-10, IL-21 and TNF-α and immunoglobulin IgG, IgM, IgA and sIgA. As shown in Figure 2A–C, compared with CON, the four tannins added to the diet significantly reduced the level of IL-1β (*p* < 0.01). CT, QT and TT can markedly significantly reduce the levels of IL-6 and TNF-α in serum (*p* < 0.01). In addition, Figure 2D shows that AT and CT can significantly increase the level of IL-2 (*p* < 0.01). There was no significant difference in the level of IL-21 in each group compared with CON (Figure 2E), but adding four tannins into the diet can significantly increase the level of IL-10 (*p* < 0.01) (Figure 2F). The IgG concentration in serum is shown in Figure 2G; feeding the diet supplemented with tannins can increase the level of IgG in serum, among which AT, CT and TT have a significant increase compared with CON (*p* < 0.01), and the levels of IgM, IgA and sIgA in the experimental groups were significantly increased in all tannin groups (*p* < 0.01) (Figure 2H–J).

Then, we compared the differences between the experimental groups; except for QT, the IL-1β level of CT and TT was significantly lower than that of AT. The IL-6 level of CT, QT and TT was significantly lower than that of AT (*p* < 0.01). In addition, the level of IL-2 in AT and CT was significantly higher than that of QT and TT (*p* < 0.01). For the effect of IL-21, the level of IL-21 in QT was significantly higher than that of TT (*p* < 0.05). Finally, the level of IL-10 in CT, QT and TT was significantly higher than that of AT (*p* < 0.01). In addition, the IL-10 level of QT was significantly higher than that of CT and TT (*p* < 0.05). The comparison between the experimental groups found that the level of IgG in AT and CT was significantly higher than that of the other two experimental groups (*p* < 0.01). The concentrations of IgM and IgA in the serum of AT were significantly higher than those of QT and TT (*p* < 0.01) but significantly lower than the levels of IgM and IgA in CT (*p* < 0.05). The IgA level of QT is significantly higher than that of TT (*p* < 0.05). The sIgA level of CT is significantly higher than that of AT, QT and TT (*p* < 0.01).

### 3.5. Intestinal Mucosal Barrier and Liver Function Evaluation

**Figure 3:**
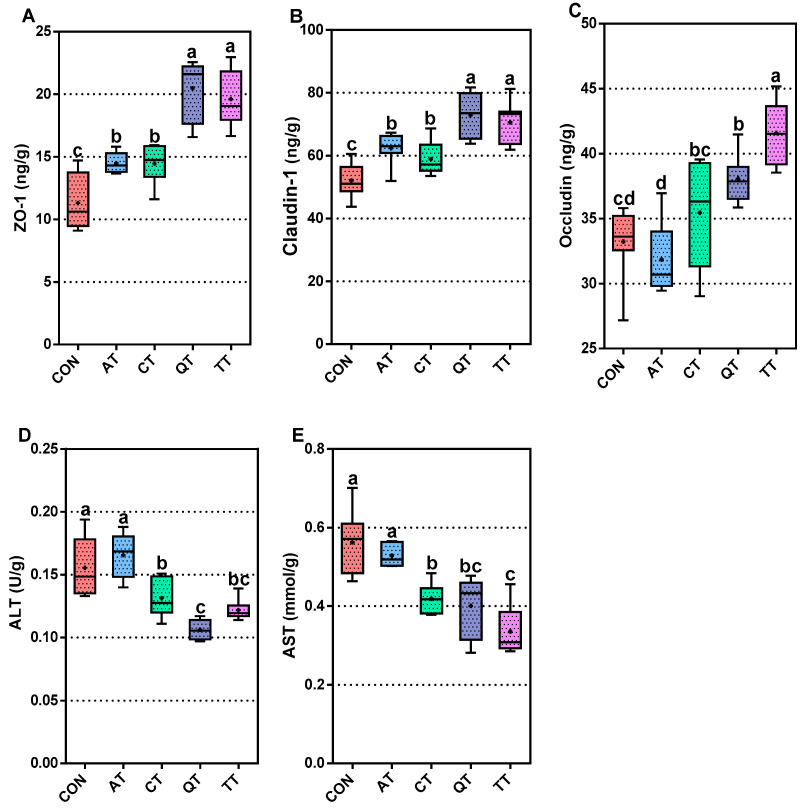
Effects of adding different tannins to the diet on the intestinal mucosal barrier and liver function. (**A**) Zonula occludens-1, ZO-1; (**B**) claudin-1; (**C**) occludin; (**D**) alanine aminotransferase test; ALT (**E**) aspartate aminotransferase test; AST. Different letters (a, b, c and d) indicate significant differences (*p* < 0.05), while the same letter indicates no significant differences (*p* > 0.05). CON, control; AT, *Acacia mearnsii* tannin; CT, *Castanea sativa* tannin; QT, *Schinopsis lorenzii* tannin; TT, *Caesalpinia spinosa* tannin.

The effects of the dietary supplementation of different tannins on the intestinal mucosal barrier and liver function of broilers were evaluated by measuring the ZO-1, claudin-1 and occludin in the jejunal mucosa and ALT and AST in serum. As shown in Figure 3A–C, compared with CON, adding four tannins to the diet can significantly increase the levels of ZO-1 and claudin-1 in the intestinal mucosa (*p* < 0.01); the concentration of occludin also significantly increased in QT and TT (*p* < 0.01). Moreover, from Figure 3D,E, we can obtain the concentrations of the ALT and AST in serum. The concentrations of ALT and AST in CT, QT and TT were significantly reduced (*p* < 0.01).

Similarly, we compared the concentrations of ZO-1, claudin-1 and occludin in each experimental group and found that the levels of ZO-1 and claudin-1 in QT and TT were significantly higher than those in AT and CT (*p* < 0.01). The results of occludin showed that the concentration in TT was significantly higher than that in AT, CT and QT (*p* < 0.01); and the occludin level of CT and QT was significantly higher than that of AT (*p* < 0.05). Finally, the concentration of ALT in QT was the lowest, which was significantly lower than that of AT and CT (*p* < 0.01). The ALT concentration of CT was significantly lower than that of AT (*p* < 0.01). In addition, TT had the lowest concentration of AST, which was significantly different from that of AT and CT (*p* < 0.05). In addition, the concentration of AST in AT was significantly higher than that in CT and QT (*p* < 0.01).

### 3.6. Assessment of Small Intestine Morphology

**Figure 4:**
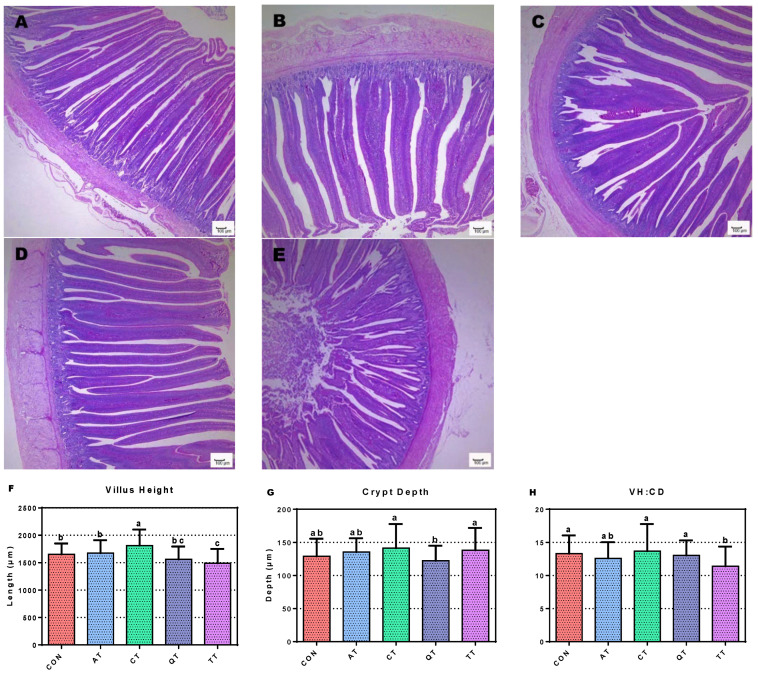
Photomicrograph of the small intestine: jejunum stained with hematoxylin and eosin stain. (**A**) CON; (**B**) AT; (**C**) CT; (**D**) QT; (**E**) TT; (**F**) villus height; (**G**) crypt depth; (**H**) ratio of villus height to crypt depth. Different letters (a, b and c) indicate significant differences (*p* < 0.05), while the same letter indicates no significant differences (*p* > 0.05). CON, control; AT, *Acacia mearnsii* tannin; CT, *Castanea sativa* tannin; QT, *Schinopsis lorenzii* tannin; TT, *Caesalpinia spinosa* tannin.

The effects of adding four tannins on the jejunum morphology of broilers are shown in Figure 4. The VH of CT was significantly longer than that of CON (*p* < 0.05), while TT was significantly lower than CON (*p* < 0.05). Compared with CON, the VH of AT showed an upward trend, while QT showed a downward trend. The comparison results between the experimental groups showed that the VH of CT is significantly longer than that of the other treatment groups (*p* < 0.01). Furthermore, QT significantly decreased CD compared to CT and TT (*p* < 0.05). Finally, the jejunal villus height to crypt depth ratio (VH/CD) in broilers showed that TT significantly reduced VH/CD compared to CON (*p* < 0.01), while the other treatment groups were not significantly different from CON, but CT showed an upward trend.

### 3.7. Effects of Different Tannins on Intestinal Flora in Healthy Subjects

**Figure 5:**
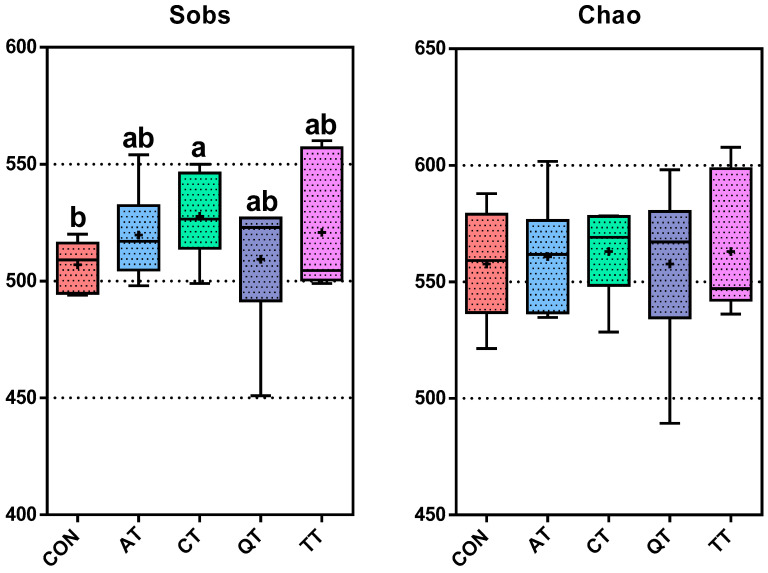

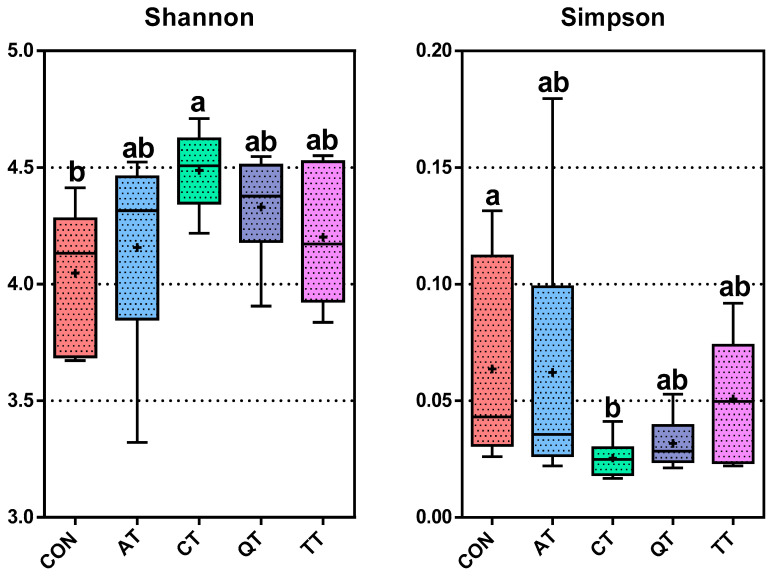
Alpha diversity analysis of gut microbial abundance (Sobs and Chao indices) and diversity (Shannon and Simpson indices) from different groups (*n* = 6/group); statistical significance was determined by Mann–Whitney U test for two-group comparisons. Different letters (a and b) indicate significant differences (*p* < 0.05), while the same letter indicates no significant differences (*p* > 0.05). CON, control; AT, *Acacia mearnsii* tannin; CT, *Castanea sativa* tannin; QT, *Schinopsis lorenzii* tannin; TT, *Caesalpinia spinosa* tannin.

**Figure 6:**
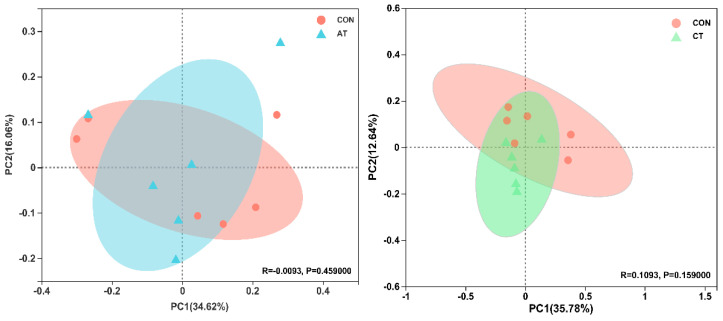

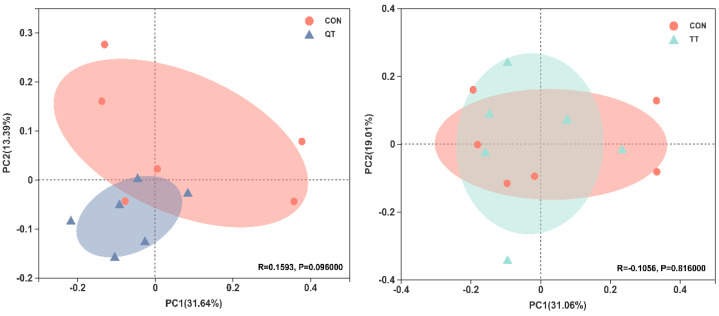
Principal coordinate analysis (PCoA) plot based on Bray−Curtis distance of the gut microbiota composition at the OTU level from different groups (*n* = 6/group). Pairwise comparisons using the analysis of similarities (ANOSIM) test. CON, control; AT, *Acacia mearnsii* tannin; CT, *Castanea sativa* tannin; QT, *Schinopsis lorenzii* tannin; TT, *Caesalpinia spinosa* tannin.

In order to accurately assess and compare the effects of four plant-derived tannins on the gut microbiota of broilers, we performed bacterial 16S rDNA amplicon sequencing of the cecal contents of all groups, and a total of 982 OTUs were detected with a similarity of 97%. The various exponential dilution curves eventually flattened, indicating that the data were sufficient for further analysis (Figure S1). To determine the richness and diversity of gut microbiota, we assessed the alpha diversity using the Sobs, Chao, Shannon and Simpson indices. As shown in Figure 5 and Table S1, the Sobs and Shannon indices of CT significantly increased compared with CON (*p* < 0.05), the Simpson index also decreased significantly (*p* < 0.05), but the Chao index did not increase significantly. Except for CT, the other three treatment groups showed no significant difference in diversity index with CON, but their Sobs, Chao and Shannon indices showed an overall upward trend, while their Simpson index showed an overall downward trend. We also performed principal coordinates analysis (PCoA) based on the Bray–Curtis distance to demonstrate the bacterial community differences between each group more clearly. As shown in Figure 6, the addition of CT and QT greatly changed the structure of the gut microbiome of broilers, compared to other samples that showed little difference with CON. This is consistent with the results presented by the alpha diversity index.

**Figure 7:**
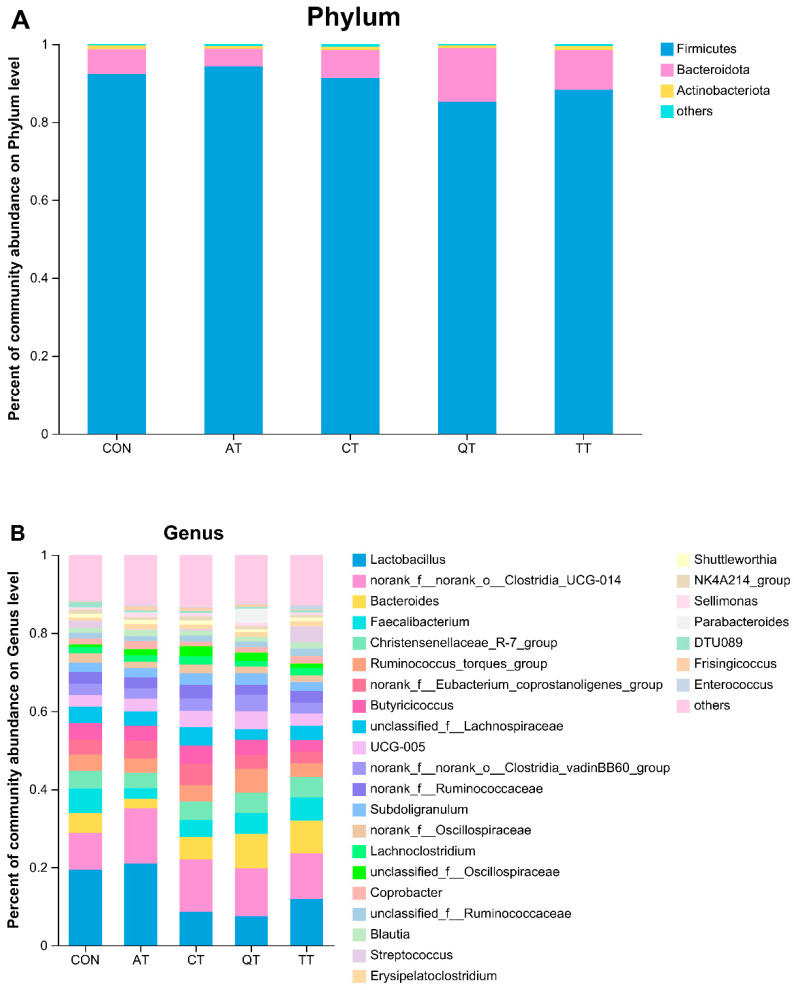
Bacterial community composition of broiler gut microbes at (**A**) phylum level and (**B**) genus level. CON, control; AT, *Acacia mearnsii* tannin; CT, *Castanea sativa* tannin; QT, *Schinopsis lorenzii* tannin; TT, *Caesalpinia spinosa* tannin.

Next, to investigate the effect of five tannins on the composition of broiler gut microbial communities, we mainly analyzed the bacterial taxonomy at the phylum and genus levels. As shown in Figure 7A, Firmicutes, Bacteroidota and Actinobacteriota were dominant at the phylum level. AT increased the abundance of Firmicutes but decreased the abundance of Bacteroidetes, whereas the remaining three tannins had the opposite effect (Table S2); TT increased the abundance of Actinobacteriota, while the three other tannins reduced its abundance. The difference at the phylum level was not significant compared to CON (*p* > 0.05), but *Firmicutes* in QT were significantly lower than those in AT (*p* < 0.05), while Bacteroidota was significantly higher in QT than in AT (*p* < 0.05). The taxonomic composition at the genus level is shown in Figure 7B, with a total of 30 genera annotated. We compared the top 15 genera in abundance and found that all four tannins increased the abundance of *o_Clostridia_UCG-014*, among which AT had a significant increase compared with CON (*p* < 0.05), while the difference of the other three tannins was not significant (*p* > 0.05). In addition, *UCG-005*, *o_Clostridia_vadinBB60_group* and *Subdoligranulum* also increased in four treatment groups (*p* > 0.05). The abundance of *Faecalibacterium* and *f_Oscillospiraceae* decreased after the addition of tannins (*p* > 0.05). AT increased the abundance of *Lactobacillus* and decreased the abundance of *Bacteroides* and *Christensenellaceae_R-7_group* (*p* > 0.05), while the other three tannins decreased the abundance of *Lactobacillus* and increased the abundance of *Bacteroides* and *Christensenellaceae_R-7_group* (*p* > 0.05). *Lactobacillus* was significantly reduced in CT and QT compared with CON (*p* < 0.05). In addition, we also found that only CT increased the abundance of *f_Lachnospiraceae* and *f_Ruminococcaceae*, while the other three tannins decreased both genera, but none of these differences were significant compared to CON (*p* > 0.05). Finally, *f_Eubacterium* was increased in the AT and CT groups (*p* > 0.05), *Butyricicoccus* was increased in CT (*p* > 0.05), and *Lachnoclostridium* was enriched in CT and TT (*p* > 0.05) (Figure S2).

**Figure 8:**
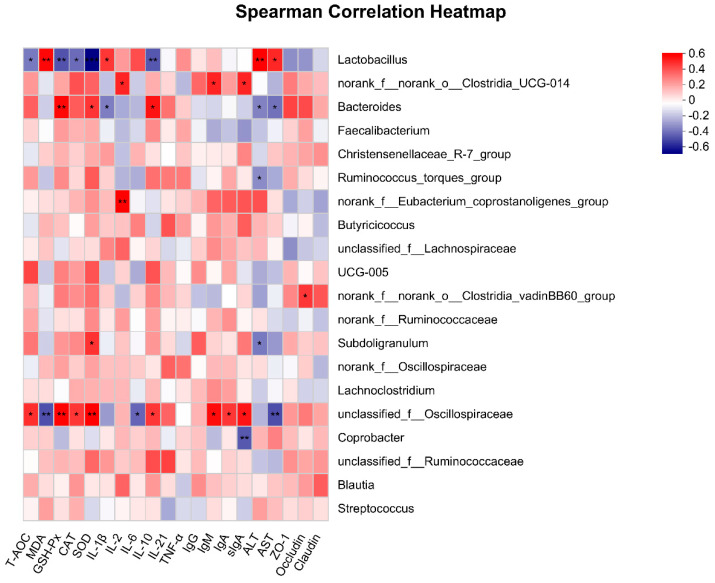
Heatmap showing Spearson correlation analysis between antioxidant-related and immune-related indicators with bacterial genera, *p* < 0.05. The red blocks represent positive correlations, and the blue blocks represent negative correlations. The colors’ shades indicate the strength of the correlations. * *p* < 0.05, ** *p* < 0.01, and *** *p* < 0.001 compared with CON group.

After understanding the bacterial community structure, we selected the top 20 species in total abundance at the genus level and performed Spearson correlation analysis with antioxidant-related and immune-related indicators to determine the potential relationship between the main microbiota with broiler antioxidant capacity and immune performance. As shown in Figure 8, nine genera showed a significant correlation with the antioxidant capacity and immune performance of broilers. *Lactobacillus* is mainly related to antioxidant capacity. It had a very significant negative correlation with antioxidant enzymes and a positive correlation with MDA (*p* < 0.01). In addition, *Lactobacillus* was also positively correlated with the production of ALT and AST (*p* < 0.01). Contrary to *Lactobacillus*, *Bacteroides* showed a significant positive correlation with the production of antioxidant enzymes (*p* < 0.05) and a significant negative correlation with ALT and AST (*p* < 0.05). We also observed that *f_Osillospiraceae* showed a significant positive correlation with the production of antioxidant enzymes (*p* < 0.01) and a significant positive correlation with serum immune factors (*p* < 0.05). *Clostridia_UCG-014* was significantly positively correlated with serum immune factor production (*p* < 0.05). *Ruminococcus* and *Subdoligranulum* were negatively correlated with ALT (*p* < 0.05). *Clostridia_vadinBB60* had a positive effect on the production of occludin (*p* < 0.05).

**Figure 9:**
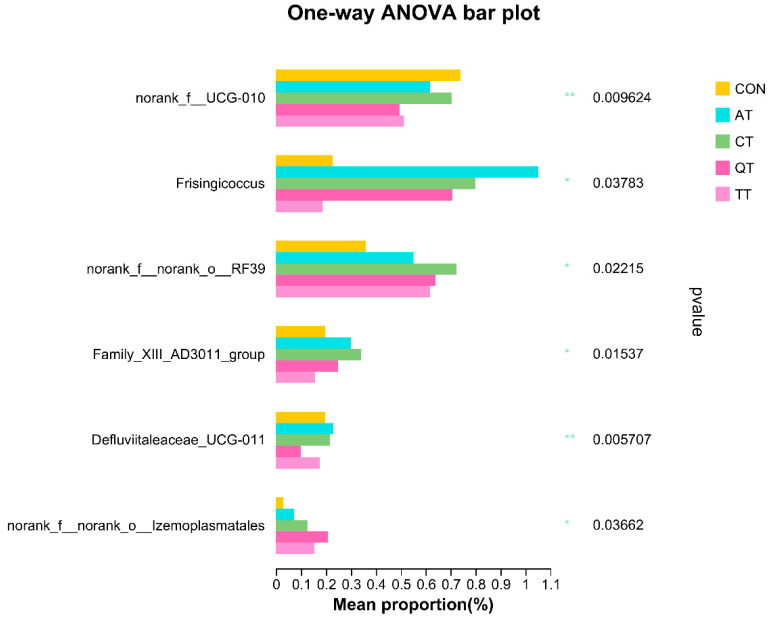
Significant test of group differences based on one-way ANOVA test. The right of the bar is the *p*-value; * *p* < 0.05, ** *p* < 0.01. CON, control; AT, *Acacia mearnsii* tannin; CT, *Castanea sativa* tannin; QT, *Schinopsis lorenzii* tannin; TT, *Caesalpinia spinosa* tannin.

In order to understand the effects of the four tannins on the gut microbial structure of broilers more specifically, we used the one-way ANOVA test to assess the statistically significant differences between the dominant bacterial genera in the gut microbiota of broilers fed different tannins (Figure 9). We found that the genera showing abundance differences in different groups of microbial communities were *f__UCG-010*, *Frisingicoccus, o__RF39, Family_XIII_AD3011_group*, *Defluviitaleaceae_UCG-011* and *o__Izemoplasmatales*. Compared with the control group, all four tannins decreased the abundance of *norank_f__UCG-010* and increased the abundance of *Frisingicoccus*, *o__RF39* and *o__Izemoplasmatales*. Except for TT, the other three tannins increased the abundance of *Family_XIII_AD3011_group*. Then, we used the Wilcoxon rank-sum test to assess the significance of the differences at the same time, and the results are shown in Figure S3. The results showed that AT and CT can significantly enriched *Frisingicoccus* in the intestinal of broilers (*p* < 0.05). In addition to this, *Negativibacillus*, *Anaerostipes, Anaerofilum* and *Catenibacillus* were significantly enriched in AT (*p* < 0.05), while CT was significantly enriched for *Clostridia_UCG-014*, *f_Oscillospiraceae*, *o__RF39* and *Oscillibacter* (*p* < 0.05). QT significantly enriched *Parabacteroides*, *f_Oscillospiraceae* and *o_Izemoplasmatales* (*p* < 0.05) and reduced enrichment of *f__UCG-010* and *Defluviitaleaceae_UCG-011* (*p* < 0.05). Finally, *o_RF39*, *Lactococcus* and *Glutamicibacter* were significantly enriched in the gut of TT-fed broilers (*p* < 0.05), while the enrichment of *f__UCG-010* and *GCA-900066575* was decreased in TT (*p* < 0.05).

## 4. Discussion

Previous studies have explored the improvement of animal nutrition and biological functions by adding plant tannins to animal diets. Tannic acid has been successfully used as a feed additive for ruminants to reduce protein degradation in the rumen due to its positive effect on rumen fermentation [29], thereby increasing protein utilization and ultimately improving animal production efficiency [30]. Tannins are “anti-nutritional factor” for monogastric animals and poultry [31], and a better application method of tannins in feeding monogastric animal and poultry has not been found yet; however, some research showed that the addition of tannins in the diet can have a positive effect on the growth performance and antioxidant activity of livestock [32,33]. However, such beneficial effects are controlled by a variety of factors, such as the concentration added to the diets, the structure of the compound, the source of tannin extraction, etc. [34,35]. In addition, most experiments focus on the impact of tannins on the growth performance or biological functions of broilers, which are concentrated on a specific tannin. Comparative research between different sources of tannins and their effects on the regulation of intestinal flora in healthy broilers is lacking. Therefore, in this study, tannins extracted from four plants were selected and added to broiler diets to investigate the effects of different sources of tannins on broiler growth performance, antioxidant capacity, immunity and gut microbiota and compare their effects on broilers.

Studies showed that adding low-dose tannins to broilers’ diets has the effect of improving growth performance [36]. When adding doses of 7 g/kg, the growth of broilers was suppressed and decreased [37], and a higher dose often showed anti-nutrition or even toxic effects [38,39]. Moreover, the focus of our research is not on the composition and content of tannins. Instead, we take the different tannin extracts as a whole to then evaluate and compare the effect of different sources of tannin extracts on broilers. Therefore, we uniformly set the addition amount of the four samples to 0.6 g/kg in this study. In addition, we then recorded various growth indicators of broilers during the experiment. The results showed that the addition of tannins does not have a significant effect on the body weight of broilers. Similarly, the changes in the ADG, ADFI and FCR of broilers after tannin supplementation were not significant. In addition, the cecum length of the broilers in each experimental group increased, but the increase effect was not significant. The aforementioned results indicate that after adding tannins, the growth performance of the broilers in each experimental group did not change significantly, which was consistent with the findings of Jamroz et al. and Tonda et al. [40,41]. Although adding an appropriate number of tannins to the broiler diet has an impact on the growth performance of broilers, the concentration does not have much impact if the concentration is too low, so the specific concentration range needs to be further explored in subsequent studies. Moreover, the ADG of broilers after adding tannins was decreased, suggesting that adding tannins to the diet reduces feed intake in broilers, which may be related to the palatability of the diet after adding tannins. In addition, the results showed that the FCR of AT and QT was higher than CT in the broilers’ growth stage, and ADG was lower than CT. The effects of TT on ADFI, ADG and FCR were inferior to those on the other groups in the growth period. These differences resulted from the same basal diets being supplemented with the same levels of tannins; AT and QT are condensed tannins, while CT and TT are hydrolyzable tannins. From the results, we cannot infer that hydrolyzable tannins have a better effect on the growth of broilers than condensed tannins, since TT, as a hydrolyzed tannin, has a worse effect on the growth performance of broilers than AT and QT. This indicates that the chemical classes of tannins may have no impact on the growth performance of broilers, but the source of tannins has an impact on the growth performance of broilers. These results differ from previous studies [42,43].

Oxidative stress plays a pathogenic role in inflammatory diseases [44], so it is extremely important to compare and evaluate the effects of the different sources of tannins on the antioxidant capacity of broilers. After adding tannins to the diet, the T-AOC of broilers was significantly increased; the concentrations of SOD, GSH-Px and CAT in serum were increased; and the levels of MDA were decreased. These results were consistent with Samuel et al. [45] and Dong et al. [46]. Elevated levels of antioxidant enzymes and decreased MDA indicate that the dietary supplementation of four plant tannins improved the antioxidant capacity of broilers. As a phenolic compound, tannin has a strong antioxidant capacity like other polyphenols, which is related to the existence of a phenolic ring in the tannin structure [47,48]. Furthermore, we observed that the effect of AT on T-AOC and SOD was significantly lower than that of other tannins, while the level of MDA in serum was significantly higher than that of other groups. Studies have shown that the antioxidant activity of polyphenols can be attributed to their phenolic ring structure [49], and the configuration and number of hydroxyl groups can significantly affect their antioxidant activity [50,51]. Based on this, we speculate that AT may have a lower number of phenolic rings than the other three plant tannins, reducing its ability to scavenge free radicals and chelate metals [52,53], which ultimately shows a weaker antioxidant capacity. Interestingly, we found that AT only significantly enhanced the activity of SOD but not the activity of GSH-Px and CAT. This indicates that AT may not stimulate GSH-Px- and CAT-related pathways and has no upregulation effect on GSH-Px and CAT, which needs further research and demonstration. From the perspective of T-AOC, CT and QT both have excellent antioxidant properties in broilers. Furthermore, CT has the best effect of enhancing antioxidant enzymes, and its scavenging ability for MDA is also the best among the four tannins, which is basically the same between the two; QT and TT are second only to CT in enhancing antioxidant enzymes, but the effect of TT on MDA is slightly less than that of QT; and, finally, although AT has good antioxidant activity, it is far behind the other three tannins. Both AT and QT are condensed tannins, but their antioxidant capacity is significantly different, which indicates that the antioxidant capacity of different natural plant tannins is not directly related to their structure, but more likely it is related to their source. This further illustrates that tannins from different sources have unique properties.

In addition to antioxidant capacity, anti-inflammatory and immunomodulatory properties are also important for the host [54]. This study proved that tannins can reduce the levels of IL-1β, IL-6 and TNF-α and increase the level of IL-10. This suggests that the addition of tannins to the diet has an immunomodulatory effect and is able to maintain the homeostasis of the immune response, which is consistent with the findings of Cao et al. [55]. In addition, the results showed that AT and CT significantly increased the level of IL-2 in serum, while QT and TT had no effect on it. The levels of IL-21 did not change significantly with the addition of tannins. Chicken IL-2 is a T cell growth factor that induces spleen T cell proliferation, increases NK cell activity and also plays a key role in inducing the production of effector cells and memory cells [56,57]. IL-21 acts as a co-stimulator of T cells in chicken and negatively regulates DCs [58]. From this, we can infer that AT and CT have a good enhancement effect on the adaptive immunity of broilers, and the same conclusion can be drawn by increasing the levels of IgG and IgM in serum by AT and CT [59], while the enhancement effect of QT and TT is relatively general or not. In our study, all four tannins significantly increased IgA in broiler serum, which is consistent with the findings of Liu et al. [60], with CT being the highest, followed by AT, QT and TT, and sIgA also showed the same result. sIgA serves as the first line of defense to protect the intestinal mucosa, and it maintains intestinal homeostasis, together with mucins [61]. This indicates that the four plant tannins also have an enhancing effect on intestinal mucosal immunity; CT had better enhancing effects than the other three, which may be related to its excellent antioxidant properties, as studies showed that enhancing the antioxidant properties of animals can affect their immunity [62]. However, the previous results showed that AT had a high rate of diarrhea without a significant increase in inflammatory factors, indicating that there was no inflammation in the broilers. Therefore, we inferred that the significant increase in serum immunoglobulin and IL-2 in AT was partly due to the activation of autoimmunity caused by diarrhea. Based on the above results, we can conclude that QT, CT and TT had the effect of reducing inflammation and improving immunoglobulin levels, and tannins may be modulating the immune performance of broilers. There were also differences in the anti-inflammatory and immunoglobulin-improving effects of the four tannins. The anti-inflammatory effects and immune-enhancing effects of CT were the best, followed by QT and TT, which were also relatively good, but the effect on immunity was limited; the last was AT, as its anti-inflammatory effect was not as good as that of the other three tannins, though its effect on improving immune factors was outstanding, partly due to the activation of autoimmunity. Hence, the improvement effect of AT on the immunity of broilers needs further research. Moreover, there was no correlation between the type of tannin and the immune-improving effect, which indicates that the differences in the effects of several natural plant tannins on broilers were, again, mainly due to their sources.

Tight junction proteins are mainly composed of cytosolic proteins zonula occludens (ZO-1 and ZO-2) and transmembrane proteins (occludin and claudin), which play a crucial role in intestinal mucosal immunity [63]. In this study, both ZO-1 and claudin-1 levels were significantly increased in the jejunal mucosa of tannin-fed broilers. The levels of occludin in each group were different. AT decreased the level of occludin in the jejunum mucosa, and CT increased the level of occludin, but the effect was not significant, while QT and TT significantly increased the level of occludin in the jejunum mucosa. Overall, feeding tannins increased the tight junction protein in the jejunal mucosa of broilers, thereby enhancing the immune function of broiler intestinal mucosa and ensuring the integrity of the intestinal barrier [64,65]. The function of occludin involves intercellular adhesion, migration and permeability; once it is mutated, reduced or absent, it can lead to increasing intercellular permeability of intestinal epithelial cells [66]. AT led to lower levels of occludin, suggesting that it may not have a beneficial effect on intestinal wall permeability, which makes it easier for pathogens such as bacteria to invade the broiler gut and trigger inflammation [67]. Our results also showed that TT increased the level of the three tight junction proteins the most; QT could significantly increase the concentrations of ZO-1 and claudin-1, which was basically consistent with the effect of TT, and the level of occludin was significantly lower than that of TT. The levels of ZO-1 and claudin-1 in the jejunal mucosa of AT is lower than those of the other treatment groups, which could be one of the reasons why the AT diarrhea rates were higher than those of the others. We also tested the ALT and AST in chicken liver to evaluate the effect of four tannins on broiler liver function. The results show that QT and TT can reduce the effect of ALT and AST, and CT also have the same effect, though the effect of the former two tannins is more effective than that of CT. However, AT showed no effect on ALT and AST. The decrease in ALT and AST indicated that tannins had an anti-hepatotoxic effect, while AT did not exhibit this effect, which may be related to its structure [68].

Dietary nutrients can modulate the small intestinal tissue morphology and digestive function of animals [69]. The villi and crypts of the small intestine play a key role in the final stages of digestion and absorption of nutrients [70]. The results of Liu et al. [71] and Tong et al. [72] showed that tannins could significantly increase VH, but they had no significant effect on CD and VH/CD. In this study, we observed that CT could increase VH significantly compared with CON. The CD and VH/CD of CT have no significant difference with CON, which is consistent with the results of Liu et al. [71] and Tong et al. [72]. We also observed a significant decrease in the VH and VH/CD in broilers after feeding TT, which indicates that TT has a negative effect on the digestive function of broilers. In addition, we did not observe other significant changes. The study of Tong et al. revealed that the effect of tannin on the intestinal morphology of broilers is closely related to the tannin concentration; thus, we speculate that the CD and the insignificant changes in the VH/CD may be related to the tannin concentration. Although the differences were not significant, the CD and VH/CD of CT have an upward trend compared with CON, while AT, QT and TT showed a downward trend. Increasing the villus height could increase the surface area of the small intestine for nutrient absorption and, thus, improve the absorption rate of dietary nutrients in the small intestine, while AT, QT and TT accomplish the opposite. Adding CT to the diets can improve the digestion and absorption of nutrients by broilers. This result corresponds well with the final body weight of broilers.

The modulation of dietary formulation could change animal gut microbial composition and digestive function [73,74]. The gut microbiome can improve the utilization rate of nutrients, reduce the colonization of pathogens, etc. [75], thereby improving the health, promoting the growth performance and enhancing the disease resistance of broilers [76]. As a potential feed additive, it is of great importance to understand the effect of tannins on the intestinal flora. Our results showed that the Sobs, Shannon and Chao indices increased, while the Simpson index decreased, after the five tannins were added to the diet, indicating that the four tannins had enhanced effects on the richness and diversity of gut microbes. Studies pointed out that the diversity and richness of gut microbes are related to the body’s immune function, and low levels of microbial diversity and types suggest that immune function may be abnormal [77], so we can infer that adding these four tannins to the diet can improve the immune performance of broilers. In addition, these four tannins are derived from different natural plants, and their effects on the intestinal flora of broilers are different. This indicates that different sources of tannins have different effects on the intestinal flora of broilers.

Next, we performed a detailed analysis of the gut microbiota at the phylum and genus levels. The phylum-level result showed that the intestinal microbiota of broiler chickens was dominated by Firmicutes, Bacteroidota and Actinobacteriota. Firmicutes and Bacteroidetes are the major components of a healthy gut microbiota, followed by Actinobacteria and Proteobacteria [78], and our results were consistent with this, suggesting that the broilers were in a healthy growth state. We found that all tannins except AT reduced the abundance of *Lactobacillus*. *Lactobacillus* in the gut was able to maintain the natural stability of the microbial community while improving broiler growth performance and utilization of feed nutrients [79,80]. The presence of *Lactobacillus* in the gut microbiota of broilers fed AT was higher, but its antioxidant-related indicators were significantly lower than those of other treatment groups. Sperson correlation analysis showed that the abundance of *Lactobacillus* was inversely proportional to the antioxidant indicators, which could be one of the reasons why the feed utilization rate of AT was higher than that of other treatment groups, but the antioxidant capacity was not as good as that of the others. *Clostridia_UCG-014* and *Eubacterium_coprostanoligenes_group* showed an upward trend after the addition of tannins, and both of them are mainly involved in the production of acids (short-chain fatty acids such as propionic acid, butyric acid, etc.) in the body [81]. This means that the four tannins can alter the production of metabolites in the body by increasing *Clostridia_UCG-014* and *Eubacterium_coprostanoligenes_group* in the intestinal tract of broilers, so the specific effects need further research. Similarly, in the correlation analysis, we found that *Clostridia_UCG-014* was significantly positively correlated with the production of serum immune factors. The abundance of AT and CT was higher than that of QT and TT, which was basically the same as the trend of their serum immune factor levels. This indicated that tannins may play a regulatory role in broiler immunity by increasing the abundance of *Clostridium_UCG-014*. In addition, *Bacteroides* have extensive glycolytic potential, since they can metabolize a variety of glycans of plant or animal origin [82], and *Bacteroidetes* are highly abundant commensal bacteria in the gut. In the present study, AT decreased the abundance of *Bacteroides*, while the other three tannins increased it. From this, we can infer that CT, QT and TT can improve the anti-inflammatory performance of broilers by increasing the abundance of *Bacteroides*, while the enhancement effect of AT is relatively weak. Correlation analysis also showed that *Bacteroidetes* were positively correlated with the level of antioxidant enzymes and anti-inflammatory factors as well as with the production of tight junction proteins. This finding further elucidates the reason why AT has fewer antioxidant and anti-inflammatory properties and a lower concentration of tight junction proteins than the other treatment groups.

*Frisingicoccus* enriched with AT and CT showed significant differences compared with the control group. The function of *Frisingicoccus* is rarely reported, but they belong to the Firmicutes, so they may contribute to VFA production in broilers. AT also significantly enriched *Negativibacillus, Anaerostipes* and *Anaerofilum*. *Negativibacillus* was often associated with the fermentation of complex carbohydrates [83], and both *Anaerostipes* and *Anaerofilum* are related to the production of short-chain fatty acids such as propionic acid and butyric acid. *f_Oscillospiraceae*, enriched in both CT and QT, is associated with the degradation of various structural carbohydrates and the production of butyrate and other substances [84]. *Clostridia_UCG-014* was only enriched in CT. According to Spearson correlation analysis, *Clostridia_UCG-014* can regulate glucose and lipid metabolism disorders, and it also shows a positive correlation with the synthesis of propionic acid and butyric acid [85]. *Candidatus_Soleaferrea* maintains intestinal homeostasis by producing metabolites that exert anti-inflammatory effects [86]. The enriched genera in QT were *Parabacteroides* and *o_Izemoplasmatales*, among which *Parabacteroides* was closely related to feed utilization efficiency [87], which is involved in the degradation of polysaccharides and the production of short-chain fatty acids [88]. There are few reports on *o_Izemoplasmatales*, and the existing studies suggested that it is involved in microbial fermentation or heterotrophic metabolic reactions in the gut [89]. TT significantly enriched *o_RF39*, *Lactococcus* and *Glutamicibacter*, according to Tymofiy Lutsiv et al. [90], while *o_RF39* showed the potential to generate acetate, which suggests that *o_RF39* may have a positive effect on acetate production. *Glutamicibacter* can produce chitinase, which can hydrolyze the cell wall of fungus, thereby enhancing the body’s ability to resist fungus [91]. Based on this, we infer that TT may have an inhibitory effect on fungus, which requires further research to prove. In addition to the aforementioned findings, we also observed that QT and TT both significantly reduced the abundance of *f_UCG-010*, and it was reported that *Lachnospira_UCG_010* can increase the production of short-chain fatty acids; moreover, *Ruminococcus_UCG-010* is related to antioxidant activity and is proportional to MDA content [92,93]. In our study, the bacterial family of *UCG-010* was not determined, which needs further research. However, either way, the reduction in UCG-010 negatively affects the growth performance of broilers.

Each source of tannins has specific effects, so further research is needed to standardize their suitability as feed additives.

## 5. Conclusions

The antioxidant capacity and immune performance of broilers are significantly improved after the addition of four tannins. AT, CT and QT have a positive effect on the growth of broilers, and CT is better than AT and QT. The protective effect of CT on the liver is not as good as that of the other three tannins, but it is better than the other three tannins at enhancing the immunity of broilers, which can be used for immune enhancement. QT and TT have better protection of the liver, so they can be used for broiler liver protection. Different tannins have different effects on intestinal microbes. The tannins derived from different plants have their own unique effects on broilers; and the structure of tannins has an impact on the growth of broilers but has little relationship with the immune performance and antioxidant function of broilers. Tannins affect broilers differently depending on the source, which provides guidance for the rational application of tannins in broiler-breeding targeted research on the biological activities of different tannins.

## Figures and Tables

**Table 1:** The basic diet information (%).

Items	Days 1–14	Days 15–28	Days 29–42
Ingredients, %			
Corn	56.80	57.36	58.88
Soybean meal	36.48	34.57	33.01
Soybean oil	1.63	3.42	3.89
NaCl	0.53	0.53	0.53
Limestone	1.40	1.22	1.05
CaHPO_4_	2.10	1.99	1.85
L-Lys•HCl	0.30	0.15	0.06
DL-Met	0.26	0.26	0.23
Choline chloride	0.10	0.10	0.10
Premix ^1^	0.40	0.40	0.40
Total	100.00	100.00	100.00
Nutrient levels ^2^			
AME, MJ/kg	12.89	13.50	13.94
Crude protein, %	21.00	20.00	20.00
Calcium, %	1.01	1.00	0.95
Available phosphorus, %	0.62	0.60	0.57
Lysine, %	1.04	0.91	0.88
Methionine, %	0.41	0.40	0.38
Threonine, %	0.74	0.70	0.70
Tryptophan, %	0.22	0.19	0.18

^1^ The premix was provided per kilogram of ration: VA, 10000IU; VD, 5200IU, VE, 40IU; VK, 8IU; VB_1_, 5IU; VB_2_, 8IU; VB_6_, 12IU; VB_12_, 2IU; biotin, 0.2 mg; nicotinic acid, 45 mg; folic acid, 0.8 mg; Fe, 100 mg, Cu, 8 mg, Zn, 100 mg, Mn, 120 mg, I, 0.3 mg, Se, 0.7 mg. ^2^ Nutrient levels were calculated values. AME, apparent metabolizable energy.

**Table 2:** Effects of adding different tannins to the diet on the weekly weight of broilers (kg).

Age	CON	AT	CT	QT	TT	*p*-Value
7 days	0.163 ± 0.005	0.163 ± 0.004	0.163 ± 0.005	0.163 ± 0.004	0.163 ± 0.002	1.000
14 days	0.504 ± 0.015	0.504 ± 0.013	0.499 ± 0.011	0.505 ± 0.008	0.495 ± 0.003	0.961
21 days	1.054 ± 0.039	1.052 ± 0.029	1.033 ± 0.030	1.063 ± 0.020	1.018 ± 0.015	0.789
28 days	1.790 ± 0.073	1.767 ± 0.052	1.744 ± 0.059	1.790 ± 0.028	1.740 ± 0.038	0.928
35 days	2.619 ± 0.104	2.585 ± 0.075	2.552 ± 0.095	2.523 ± 0.032	2.496 ± 0.050	0.803
42 days	3.370 ± 0.105	3.320 ± 0.095	3.329 ± 0.118	3.252 ± 0.047	3.157 ± 0.045	0.478
49 days	3.923 ± 0.132	3.908 ± 0.077	3.932 ± 0.125	3.837 ± 0.098	3.730 ± 0.077	0.627

CON, control; AT, *Acacia mearnsii* tannin; CT, *Castanea sativa* tannin; QT, *Schinopsis lorenzii* tannin; TT, *Caesalpinia spinosa* tannin.

**Table 3:** ADFI, ADG and FCR of broilers.

Parameters	CON	AT	CT	QT	TT	*p-*Value
ADFI (kg)	0.143 ± 0.004	0.142 ± 0.005	0.140 ± 0.005	0.139 ± 0.001	0.139 ± 0.002	0.915
ADG (kg)	0.089 ± 0.003	0.089 ± 0.002	0.090 ± 0.003	0.087 ± 0.002	0.084 ± 0.002	0.611
FCR	1.602 ± 0.020	1.586 ± 0.031	1.564 ± 0.044	1.596 ± 0.037	1.634 ± 0.016	0.632

Average daily feed intake (ADFI, kg) = total feed consumption/feeding days; average daily gain (ADG, kg) = (final body weight − initial body weight)/feeding days; feed conversion rate (FCR) = ADFI/ADG. CON, control; AT, *Acacia mearnsii* tannin; CT, *Castanea sativa* tannin; QT, *Schinopsis lorenzii* tannin; TT, *Caesalpinia spinosa* tannin.

**Table 4:** Effects of adding different tannins to the diet on diarrhea rate and cecum length of broilers (mean + SD).

Parameters	CON	AT	CT	QT	TT	*p*-Value
DR (%)	0.30 ± 0.06	0.36 ± 0.04	0.28 ± 0.04	0.25 ± 0.02	0.30 ± 0.04	0.417
CL (cm)	17.48 ± 0.61	19.12 ± 0.47	18.50 ± 0.51	17.57 ± 0.57	17.77 ± 0.77	0.251

Values in the same row with different small letters superscripts indicate significant differences between experimental groups (*p* < 0.05). DR, diarrhea rate; CL, cecum length; CON, control; AT, *Acacia mearnsii* tannin; CT, *Castanea sativa* tannin; QT, *Schinopsis lorenzii* tannin; TT, *Caesalpinia spinosa* tannin.

## Data Availability

The data presented in this study are available on request from the corresponding author. The availability of the data is restricted to investigators based in academic institutions.

## Supplementary Materials

The following supporting information can be downloaded at https://www.mdpi.com/article/10.3390/antiox12020441/s1. Figure S1: Alpha diversity index curves of microbiota in different groups. (A) Sobs index, (B) Chao index, (C) Shannon index and (D) Simpson index. CON, control; AT, *Acacia mearnsii* tannin; CT, *Castanea sativa* tannin; QT, *Schinopsis lorenzii* tannin; TT, *Caesalpinia spinosa* tannin. Table S1: Effects of adding different tannins to the diet on diversity indices (mean + SD). Table S2: Proportion of various bacterial communities at the phylum level (%). Figure S2: The abundance of the top 15 species at the genus level. Different small letter (a and b) superscripts indicate significant differences (*p* < 0.05). CON, control; AT, *Acacia mearnsii* tannin; CT, *Castanea sativa* tannin; QT, *Schinopsis lorenzii* tannin; TT, *Caesalpinia spinosa* tannin. Figure S3: The relative abundance of genera with significant difference compared with control group in each tannin group. Statistical significance was determined by Wilcoxon rank-sum test for two groups comparisons. *, compared with control group. *, *p* < 0.05; **, *p* < 0.01. (*n* = 6/group). (A) CON-AT, (B) CON-CT, (C) CON-QT and (D) CON-TT. CON, control; AT, *Acacia mearnsii* tannin; CT, *Castanea sativa* tannin; QT, *Schinopsis lorenzii* tannin; TT, *Caesalpinia spinosa* tannin. The 16S rDNA raw data were uploaded to https://ngdc.cncb.ac.cn/, and the assigned accession of the submission is CRA007808.
