# Supplementary material for: Comparison of the Effects between Tannins Extracted from Different Natural Plants on Growth Performance, Antioxidant Capacity, Immunity, and Intestinal Flora of Broiler Chickens
Source: Antioxidants (Basel). 2023 Feb 10;12(2):441. doi: 10.3390/antiox12020441 (PMC9952188; doi:10.3390/antiox12020441)
Supplement: Supplementary file 1 [file antioxidants-12-00441-s001.zip › antioxidants-2166268-supplementary.pdf]

Supplementary

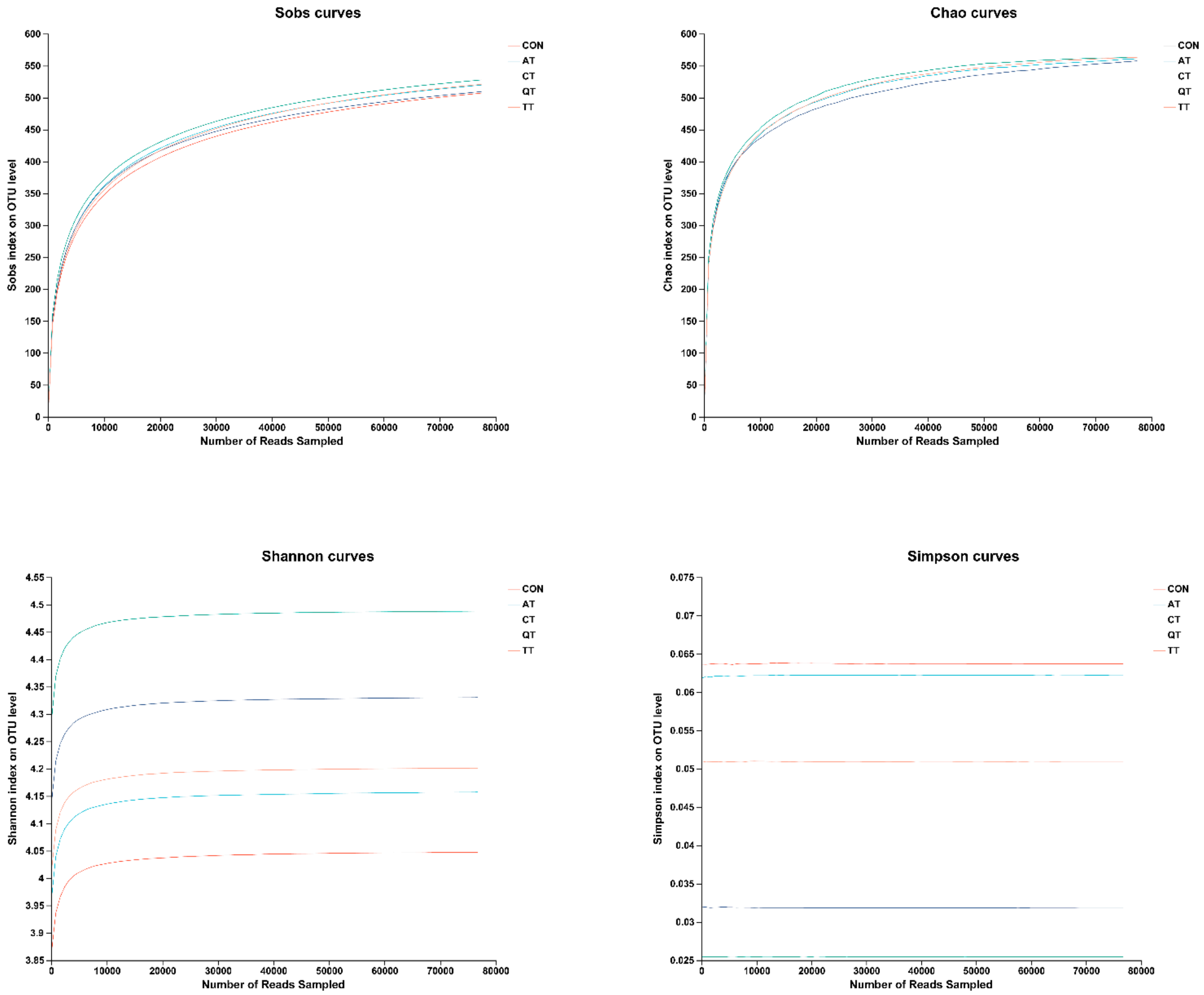

Figure S1. Alpha-diversity index curves of microbiota in different groups. (**A**)Sobs index, (**B**)Chao index, (**C**)Shannon index, (**D**)Simpson index. CON, control; AT, *Acacia mearnsii* tannin; CT, *Castanea sativa* tannin; QT, *Schinopsis lorenzii* tannin; TT, *Caesalpinia spinosa* tannin.

Table S1. Effects of adding different tannins to the diet on diversity indices (mean + SD).

Parameters	CON	AT	CT	QT	TT
Sobs	506.83±10.72^{b}	520.83±20.28^{ab}	524.50±16.81^{a}	508.17±27.46^{ab}	517.50±22.33^{ab}
Chao	549.24±24.29	561.38±30.27	563.47±15.87	556.79±31.76	563.48±41.65
Shannon	4.05±0.31^{b}	4.16±0.45^{ab}	4.48±0.17^{a}	4.33±0.23^{ab}	4.20±0.31^{ab}
Simpson	0.064±0.044^{a}	0.062±0.061^{ab}	0.026±0.009^{b}	0.032±0.011^{ab}	0.051±0.028^{ab}

Values in the same row with different small letter (a, b) superscripts indicate significant differences ($p < 0.05$). CON, control; AT, *Aca cia mearnsii* tannin; CT, *Castanea sativa* tannin; QT, *Schinopsis lorenzii* tannin; TT, *Caesalpinia spinosa* tannin.

Table S2. Proportion of various bacterial communities at the phylum level (%).

Community type	CON	AT	CT	QT	TT
Firmicutes	92.29±1.36^{ab}	94.20±1.15^{a}	91.37±1.48^{ab}	85.14±4.05^{b}	88.26±3.73^{ab}

Bacteroidota	6.37±1.37[ab]	4.57±1.05[b]	7.07±1.36[ab]	13.80±4.04[a]	8.74±1.18[ab]
Actinobacteriota	0.95±0.05[ab]	0.77±0.09[b]	0.91±0.15[ab]	0.71±0.10[b]	0.99±0.20[ab]
Others	0.39±0.08	0.46±0.13	0.65±0.28	0.35±0.04	0.54±0.16

Values in the same row with different small letter (a, b) superscripts indicate significant differences ($p < 0.05$). CON, control; AT, *Acacia mearnsii* tannin; CT, *Castanea sativa* tannin; QT, *Schinopsis lorenzii* tannin; TT, *Caesalpinia spinosa* tannin.

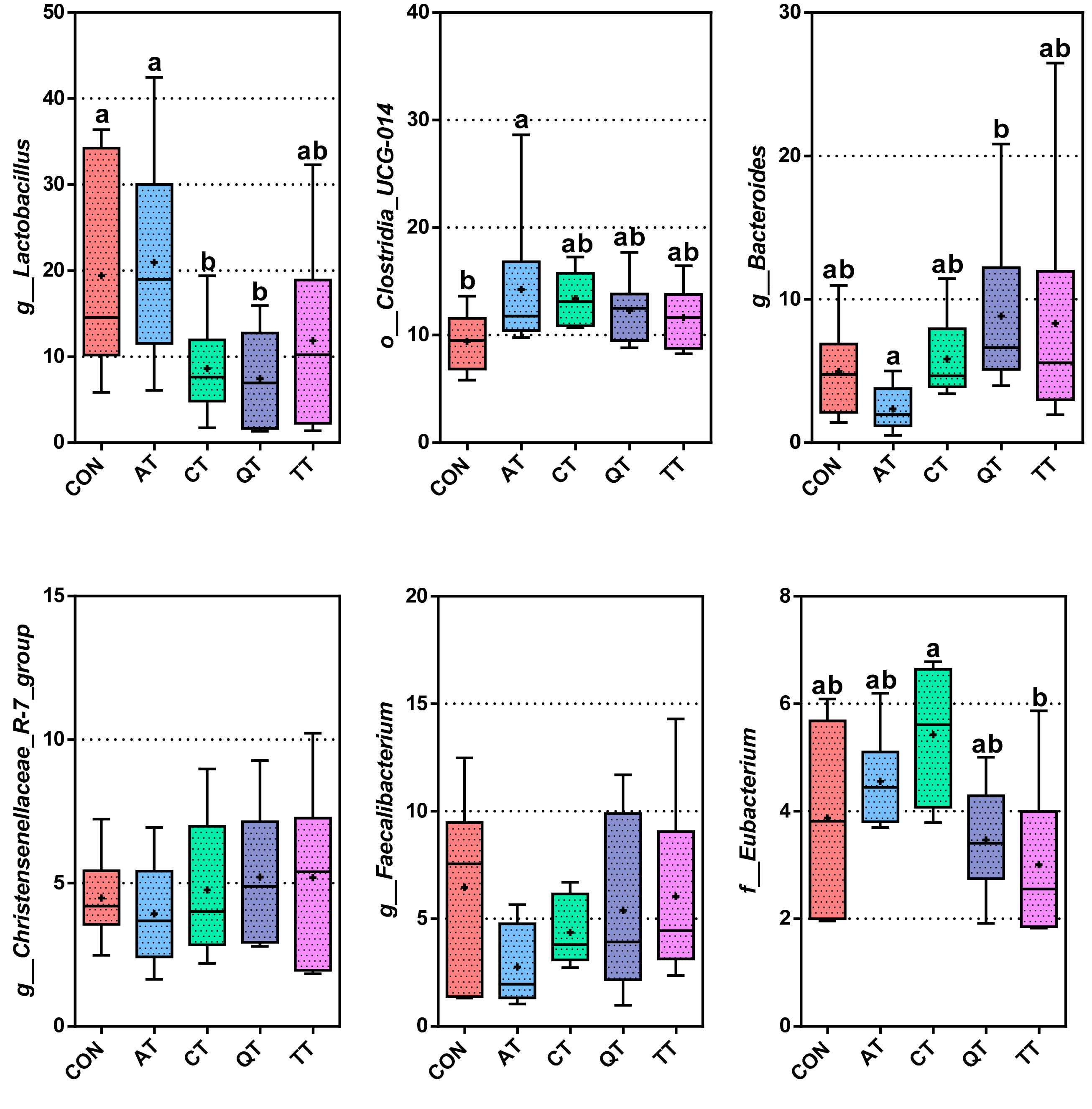

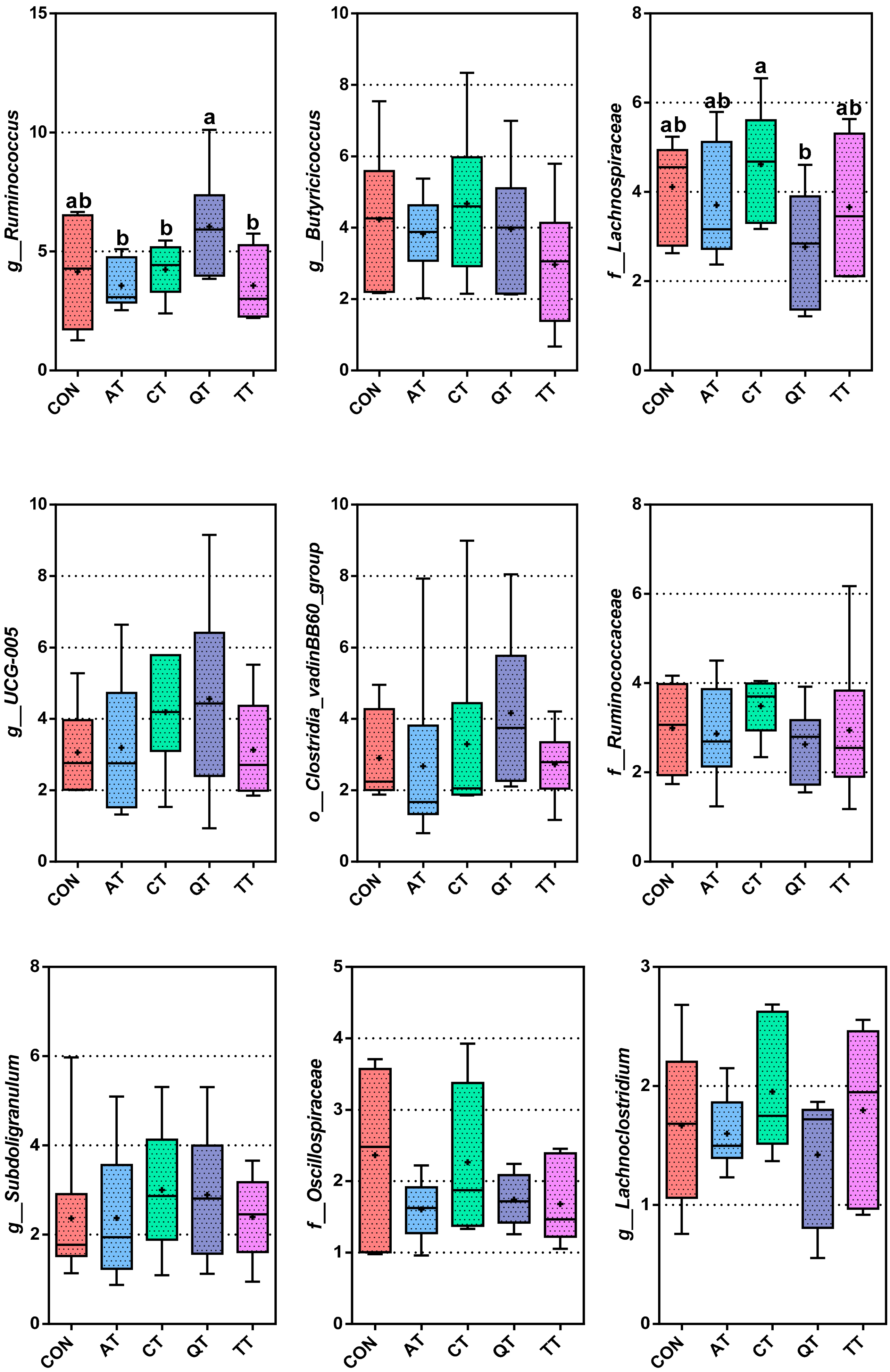

g__Ruminococcus
ab
b
b
a
b
g__Butyricicoccus
f__Lachnospiraceae
ab
ab
a
b
ab
g__UCG-005
o__Clostridia_vadinBB60_group
f__Ruminococcaceae
g__Subdoligranulum
f__Oscillospiraceae
g__Lachnoclostridium
CON
AT
CT
QT
TT

Figure S2. The abundance of the top 15 species at the genus level. Different small letter (a, b) superscripts indicate significant differences ($p < 0.05$). CON, control; AT, *Acacia mearnsii* tannin; CT, *Castanea sativa* tannin; QT, *Schinopsis lorenzii* tannin; TT, *Caesalpinia spinosa* tannin.

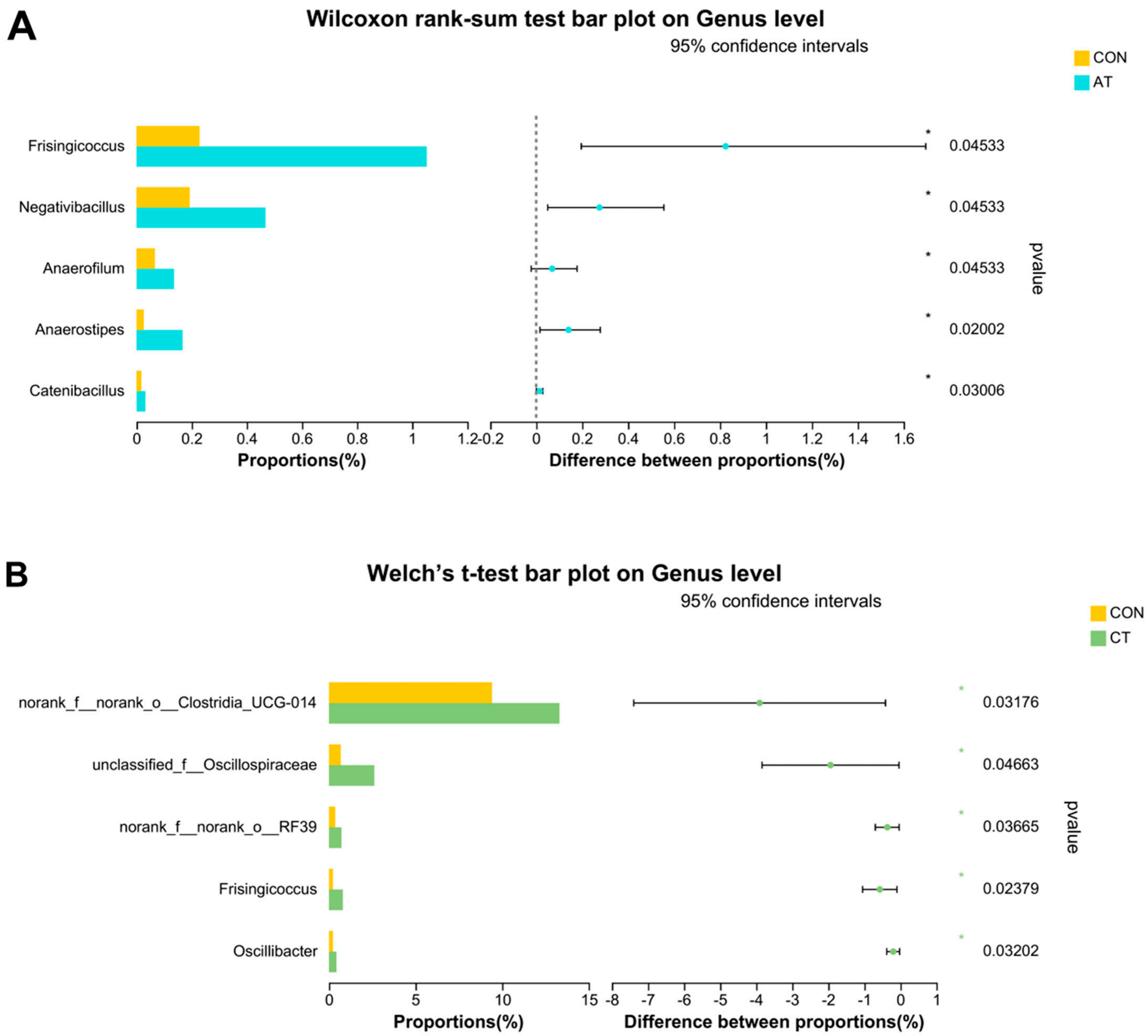

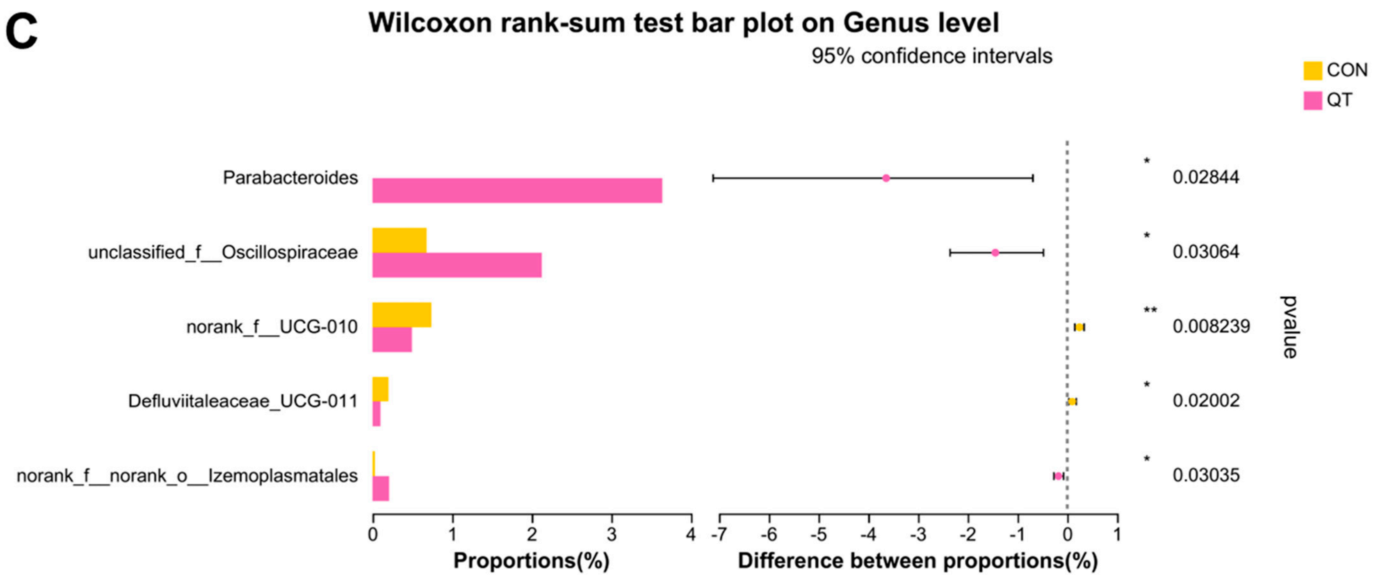

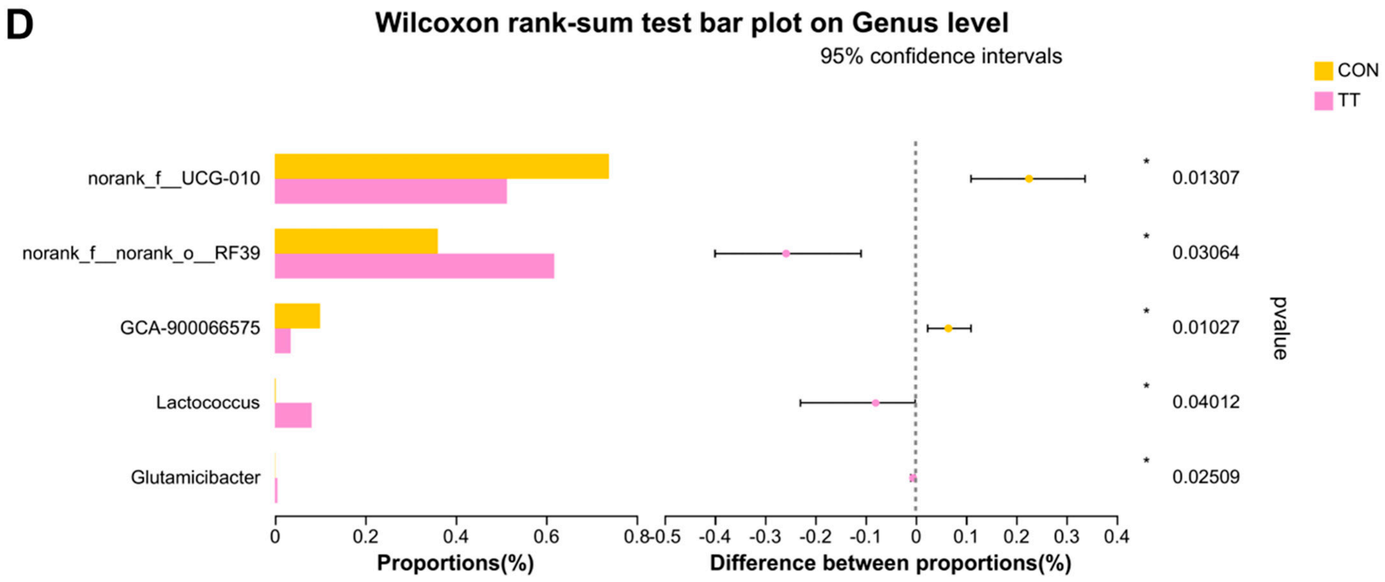

Figure S3. The relative abundance of genera with significant difference compared with control group in each tannin group. Statistical significance was determined by Wilcoxon rank-sum test for two groups comparisons. *, compared with control group. *, $p < 0.05$; **, $p < 0.01$. (n = 6/group). (**A**)CON-AT, (**B**)CON-CT, (**C**)CON-QT, (**D**)CON-TT. CON, control; AT, *Acacia mearnsii* tannin; CT, *Castanea sativa* tannin; QT, *Schinopsis lorenzii* tannin; TT, *Caesalpinia spinosa* tannin.
